# Monitoring vascular changes in renal fibrosis in mice using multimodal optical and ultrasonic imaging

**DOI:** 10.1002/btm2.70169

**Published:** 2026-08-09

**Authors:** Ruijing Zhang, Yuhang Zhang, Chuanlong Lu, Keyi Fan, Heng Wang, Qian Wang, Yijie Ning, Yaling Li, Zeyu Zhang, Xiaohua Jia, Honglin Dong

**Affiliations:** ^1^ Vascular Institute of Shanxi Medical University, Shanxi Medical University Taiyuan Shanxi People's Republic of China; ^2^ Department of Nephrology The Second Hospital of Shanxi Medical University Taiyuan Shanxi People's Republic of China; ^3^ Department of Vascular Surgery The Second Hospital of Shanxi Medical University Taiyuan Shanxi People's Republic of China; ^4^ Department of Ultrasound The Second Hospital of Shanxi Medical University Taiyuan Shanxi People's Republic of China; ^5^ Key Laboratory of Molecular Imaging of Chinese Academy of Sciences Institute of Automation, Chinese Academy of Sciences Beijing People's Republic of China

**Keywords:** chronic kidney disease, continuous microcirculation monitoring, contrast‐enhanced ultrasound, diabetic kidney disease, duplex ultrasound, near‐infrared II imaging, real‐time and wide‐field laser speckle imaging

## Abstract

Chronic kidney disease (CKD) has become one of the major diseases threatening global health, with its increasing incidence and mortality rates. Early identification of chronic kidney disease is crucial for accurate disease staging, timely intervention, and improved prognosis. However, the commonly used clinical diagnostic indicators, such as creatinine, albumin level and glomerular filtration rate, can only identify advanced CKD. Imaging examinations, such as computed tomography and magnetic resonance imaging, have limitations such as high costs and high risk of complications, and are not suitable for large‐scale screening of high‐risk populations. Renal biopsy is the gold standard for diagnosing renal fibrosis, but it is invasive. Previous studies have shown that capillary rarefaction is an early event of renal fibrosis, occurring earlier than tubular atrophy and interstitial collagen deposition. However, there is a lack of a minimally invasive, low‐cost, high‐resolution, dynamic and real‐time monitoring method for renal microcirculation that is suitable for large‐scale screening. There is an urgent need for reliable methods to screen early‐stage CKD patients. In this study, we have for the first time combined real‐time and wide‐field laser speckle imaging (RFLSI), near‐infrared II imaging (NIR‐II), duplex ultrasound (DUS) and Contrast‐Enhanced Ultrasound (CEUS) to monitor vascular changes and evaluate the progression of renal fibrosis. The combination of four imaging methods enabled the early monitoring of CKD progression by monitoring microcirculation. Among them, CEUS can detect changes in the renal microcirculation at the early stage of renal fibrosis, even earlier than the pathological damage of the kidneys. Through visualization and quantification of the progression of renal fibrosis and validation through histopathology, this study utilizes preclinical imaging to supplement clinical imaging of renal fibrosis, providing a more comprehensive method for monitoring microcirculation, which is helpful for guiding clinical decisions and providing insights into disease progression.

## 1 BACKGROUND

Chronic kidney disease (CKD) is a disease characterized by progressive loss of kidney structure or function over time, usually lasting more than 3 months.^1^, ^2^ CKD was defined according to the KDIGO 2012 guidelines as a glomerular filtration rate (GFR) below 60 mL/min/1.73 m^2^, the presence of markers of kidney injury, or both for at least 3 months, regardless of the underlying cause.^3^ Renal fibrosis (RF), characterized by excessive extracellular matrix deposition leading to scar formation, is the hallmark manifestation of different progressive forms of CKD.^4^ Diabetic kidney disease (DKD) is a major cause of morbidity and mortality in patients with diabetes and the leading cause of end‐stage renal disease worldwide.^5^ Renal hemodynamic changes, oxidative stress, inflammation, hypoxia, and hyperactive renin‐angiotensin‐aldosterone system (RAAS) are involved in the pathogenesis of DKD, and renal fibrosis plays a key role in this process.^6^, ^7^

RF, associated with excessive accumulation of extracellular matrix (ECM) proteins and myofibroblasts, is a common consequence of various progressive kidney diseases and a major process in the progression of CKD to end‐stage renal disease (ESRD).^8^, ^9^ Many pathophysiological principles of renal fibrosis are shared with other fibrotic diseases, such as cirrhosis,^10^ cardiomyopathy,^11^, ^12^ and idiopathic pulmonary fibrosis.^13^ Overall, fibrotic diseases are estimated to account for half of deaths in developed countries.^14^ The severity of renal fibrosis is the most important clinical risk factor for poor prognosis in CKD patients,^15^, ^16^ and it is also the best predictor of CKD progression.^9^ Therefore, timely and accurate diagnosis, assessment of the degree of renal fibrosis, and appropriate treatment become essential.

At present, renal biopsy is the gold standard for assessing the degree of renal fibrosis. Renal biopsy can distinguish different types of renal diseases, such as glomerulonephritis and interstitial nephritis, and provide an important basis for the etiological diagnosis of fibrosis.^17^, ^18^, ^19^, ^20^ However, it is invasive and not suitable for patients with severe renal insufficiency.^21^ In addition, techniques for the evaluation of diseased kidneys, such as computed tomography and magnetic resonance imaging, have limitations such as high cost and a high risk of complications, and are not suitable for large‐scale screening of high‐risk populations. Therefore, imaging techniques that can be rapidly detected, visualized, easily operated, and dynamically observed in real time are particularly important.

In recent years, advancements in imaging technology have opened up new possibilities for addressing this issue. Various methods such as real‐time and wide‐field laser speckle imaging (RFLSI), near‐infrared II imaging (NIR‐II), duplex ultrasound (DUS) and contrast‐enhanced ultrasound (CEUS) have gradually matured. RFLSI has been used to evaluate ophthalmic microcirculation and foot hemodynamics. RFLSI is an imaging tool capable of quantitative assessment of blood flow.^22^, ^23^, ^24^ RFLSI has the advantages of short measurement time, non‐contact, and continuous examination. Compared with NIR‐I (650–900 nm), NIR‐II (1000–1700 nm) has the advantages of deeper tissue penetration, higher spatial resolution, and reduced normal tissue scattering.^25^ Indocyanine green (ICG), a near‐infrared reagent approved by the US Food and Drug Administration (FDA), is widely used in the diagnosis of liver fibrosis, tumor resection guidance, and sentinel lymph node identification, and has great potential in vascular imaging.^26^, ^27^ DUS is generally considered to be the preferred imaging technique for assessing the degree of CKD and is important for the evaluation of internal organs.^28^ CEUS is a new imaging technology gradually developed in recent years and has gradually become the mainstream in the field of imaging. CEUS takes advantage of the strong reflective properties of microbubble contrast agents, which typically consist of a gas core and shell, under ultrasound to enhance the visualization of blood flow signals. These microbubbles can significantly improve the resolution and contrast of ultrasound images when flowing in blood, allowing clearer observation of the hemodynamic characteristics of organs and tissues.^29^, ^30^, ^31^ Sulfur Hexafluoride (SF_6_) is a safe microbubble suspension. The diameter of microbubbles is smaller than that of red blood cells, and it is harmless to the human body.^32^ After intravenous injection, SF_6_ can flow in the blood and enhance the reflection of ultrasound signals. This makes the blood vessels and tissues of the heart, liver, kidney, and other organs clearer on the ultrasound image, which helps to diagnose the disease more accurately.^33^

Despite the availability of advanced clinical modalities such as CT and MRI, their application in monitoring early‐stage renal fibrosis is constrained by either insufficient spatial resolution or potential nephrotoxicity. To address these limitations, we developed a tiered multimodal imaging framework. We have summarized the strategic advantages and limitations of our approach compared to prevalent clinical methods in Table 1. By combining optical validation (RFLSI and NIR‐II) with non‐invasive ultrasonography (DUS and CEUS), this framework ensures both rigorous experimental accuracy and a nephrotoxic‐free pathway for clinical translation.

**TABLE 1:** Advantages and limitations of various imaging modalities.

Category	Modality	Pros & Cons
Optical imaging	1. RFLSI	Pros: Extremely high temporal resolution for real‐time blood flow imaging; precise quantification of superficial microcirculation velocity; It is a label‐free imaging technique utilizing endogenous red blood cell scattering, ensuring absolutely zero exogenous toxic interference. Cons: Extremely shallow penetration depth (limited to a few millimeters); highly invasive: requiring surgical exposure.
	2. NIR‐II	Pros: Provides wide‐field, pan‐cortical dynamic mapping of global tracer kinetics (wash‐in/wash‐out rates), serving as a definitive kinetic ground‐truth; Compared to traditional NIR‐I, it exhibits extremely low tissue scattering and autofluorescence, achieving an optimal balance of penetration depth and high contrast. Cons: Relies on exogenous fluorescent probes (e.g., ICG, though non‐nephrotoxic); highly invasive: requiring surgical exposure.
Ultrasound Imaging	1. Gray‐scale US	Pros: Non‐invasive, safe, and low cost; enables point‐of‐care (POC) real‐time monitoring; excellent longitudinal follow‐up capability without radiation burden or contrast agents. Cons: Qualitative and severely lagged; completely ineffective for monitoring early microvascular/hemodynamic changes induced by fibrosis.
	2. DUS	Pros: Non‐invasive, safe, and low cost; enables POC real‐time monitoring; excellent longitudinal follow‐up capability without radiation burden or contrast agents. Quantitatively reflects macro‐hemodynamics (Resistive Index, RI). Cons: Millimeter‐level spatial resolution is insufficient for visualizing capillary networks; susceptible to operator‐dependent Doppler angle variations.
	3. CEUS	Pros: Non‐invasive, safe, and low cost; enables POC real‐time monitoring; excellent longitudinal follow‐up capability without radiation burden; The core of the “one‐stop” suite. Elegantly maps both the structural degradation of the vascular tree and localized functional micro‐perfusion deep within tissues; Microbubbles possess pure blood‐pool properties (strictly intravascular) without tissue extravasation, accurately reflecting true perfusion, Eliminated via pulmonary exhalation, posing zero metabolic burden on the kidneys. Cons: Requires intravenous microbubble injection; lacks the pan‐cortical macroscopic view provided by invasive optical modalities.
Magnetic Resonance Imaging (MRI)	1. T1/T2 MRI	Pros: Excellent soft‐tissue contrast; clearly displays macroscopic renal anatomy, cortical thickness, and structural lesions without radiation. Cons: Lacks functional sensitivity; completely unable to reflect dynamic blood flow or detect early cellular‐level fibrosis; high cost.
	2. CE‐MRA	Pros: Morphological gold standard for diagnosing macro‐vascular lesions with clear 3D reconstruction. Cons: Gadolinium agents are prone to leakage in fibrotic tissues and pose a fatal risk of Nephrogenic Systemic Fibrosis (NSF) in severe CKD/DKD patients; Limited spatial resolution for microvascular visualization; high cost.
	3. DWI	Pros: Non‐invasive and contrast‐free; reflects extracellular matrix changes via water diffusion (ADC). Cons: Low spatial resolution. Highly susceptible to motion artifacts (respiration/heartbeat), and capillary perfusion interference requires complex modeling, leading to poor reproducibility; high cost.
	4. DCE‐MRI	Pros: Provides extensive quantitative data on tissue micro‐perfusion kinetics with excellent soft‐tissue contrast. Cons: Gadolinium agents are prone to leakage in fibrotic tissues and pose a fatal risk of Nephrogenic Systemic Fibrosis (NSF) in severe CKD/DKD patients; high cost.
Computed Tomography (CT)	1. Non‐contrast CT	Pros: Extremely rapid scanning speed; superior spatial resolution compared to conventional ultrasound; highly sensitive to calcifications. Cons: Poor soft‐tissue contrast, unable to distinguish early fibrotic cortical changes; carries ionizing radiation risk; lacks functional blood flow data.
	2. CTA	Pros: Rapid acquisition and excellent 3D spatial resolution for macro‐vasculature. Cons: Dual risk of ionizing radiation and lethal Contrast‐Induced Nephropathy (CIN), as iodine agents directly damage compromised kidneys.
	3. Micro‐CT	Pros: Ultimate 3D spatial resolution (sub‐micrometer) for animal research, capable of creating extremely detailed vascular casting models. Cons: Incapable of in vivo dynamic monitoring (usually requires euthanasia and vascular perfusion); lacks real‐time hemodynamic functional data; zero potential for clinical translation.

To systematically validate this framework, we established mouse models of CKD and DKD. Using our multimodal approach, we dynamically monitored renal microcirculation during fibrosis progression (Figure 1). Disease progression was visualized and quantified through microcirculatory alterations, which were rigorously validated against histopathology. By comprehensively correlating these imaging and histological findings, this study establishes a highly sensitive and dynamic in vivo strategy for renal microvascular assessment. Ultimately, this tiered approach provides novel insights into the early detection and continuous monitoring of disease severity in CKD, laying a solid foundation for the safe clinical translation of advanced imaging techniques in nephrology.

**FIGURE 1:**
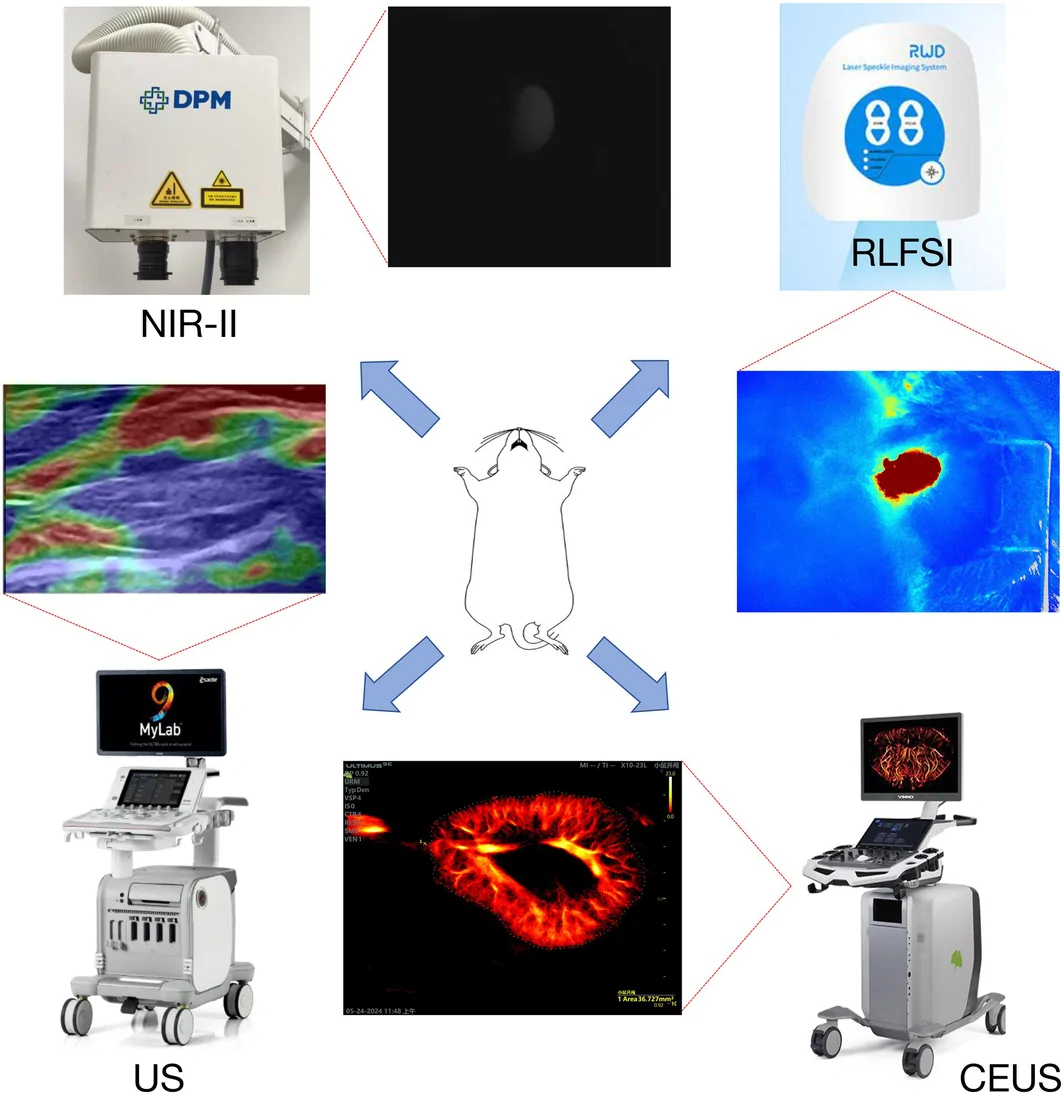
The schematic diagram of renal microcirculation imaging based on RFLSI, NIR‐II, DUS, and CEUS.

## 2 METHODS

### 2.1 Animals and experimental cohort design

Eight‐week‐old SPF‐grade male C57 mice and six‐week‐old SPF‐grade male db/db mice and db/m mice were all purchased from Nanjing Junke Biotechnology Co., Ltd. All experimental animals were raised in an environment with controlled lighting (12:12 light–dark cycle), temperature control (22°C), and humidity control (50%), and were fed with standard feed. The CKD group was freely provided with pure water, while the DKD group was freely given acidic water. All animal experiments were approved by the Ethics Committee of the Second Hospital of Shanxi Medical University (No. DW2023046).

To accommodate the differing invasiveness of the imaging modalities and to ensure statistical rigor, a total of 55 mice were utilized in this study, meticulously divided into two parallel experimental cohorts: (1) Longitudinal Ultrasound Cohort: For the non‐invasive modalities (DUS and CEUS), the same mice were longitudinally monitored across the designated disease progression time points. This cohort consisted of 20 mice (*n* = 5 per subgroup). This repeated‐measures design inherently minimized individual variability. Crucially, to prevent statistical confounding between distinct genetic or procedural baselines, the multiparametric mixed‐model statistical analysis was specifically performed on the longitudinal tracking data of the diseased cohorts to evaluate progression trajectories.^34^ (2) Cross‐sectional Optical and Histology Cohort: Because optical imaging (RFLSI and NIR‐II) requires surgical exposure of the kidneys (a terminal procedure), different subsets of mice were utilized for each specific time point. Following optical imaging, these same mice were immediately euthanized to harvest kidneys for spatially matched histopathological analysis. This cohort required a total of 35 mice, allocating *n* = 4–5 independent mice per specific time point for each disease model and control group.

### 2.2 Mouse model of CKD induced by 5/6 nephrectomy

C57BL/6J mice (male, 8 weeks old) were purchased from Nanjing Junke Bioengineering Co., LTD. After anesthesia with ether inhalation, they were placed on a heating blanket to maintain body temperature. The backs of the mice were depilated and sterilized for skin preparation. In the first stage, the skin and muscle were cut at 0.5 cm of the left back paraspinal and 0.5 cm of the lower edge of the ribs, and the kidney was visible. The whole left kidney was carefully exposed, the surrounding fascia and connective tissue were separated, and the upper and lower 1/3 of the left kidney tissue were removed. The skin and muscle were cut at 0.5 cm of the right back paravertebral and 0.5 cm of the lower edge of the ribs, and the whole right kidney was carefully exposed. The surrounding fascia and connective tissue were separated. The proximal and distal ends of the renal pedicle were ligated, and then the right kidney was cut between the two ligation points. The skin was closed with discontinuous absorbable sutures. After the mice woke up, they were put back to the clean cage and then returned to the feeding room, and the state of the mice was observed regularly and recorded. The control group also underwent a two‐step procedure, but without ligation of the left kidney or removal of the right kidney.

### 2.3 Mouse model of DKD

Male db/db mice (a well‐established murine model of type 2 diabetes harboring a spontaneous leptin receptor mutation) and their age‐matched heterozygous, non‐diabetic littermates (db/m), aged 6 weeks, were purchased from Nanjing Junke Bioengineering Co., Ltd. (Nanjing, China). The db/m mice served as the normal control group. All mice were maintained on a standard breeding diet with access to acidified water ad libitum. Their blood glucose levels were continuously monitored. Due to their genetic predisposition, these db/db mice spontaneously developed hyperglycemia and hallmark features of early DKD by 6 weeks of age.

### 2.4 RFLSI imaging and analysis

Mouse kidneys were imaged using a real‐time wide‐field laser scattered flow imaging system (SIM BFI ZOOM Pro, SIM Opto‐Technology Co., Ltd., Wuhan, China). Before RFLSI imaging, the anesthesia of mice was done by a small animal anesthesia machine (TAIJI‐IE, RWD Life Science Co., LTD., China) using isoflurane. All imaging mice were placed supine on the RFLSI imaging platform after removing abdominal hair, exposing the kidneys and fully covering the surrounding areas of the kidneys with defatted cotton balls to reduce the influence of blood flow in the perirenal organs on renal imaging. Kidneys of model and control mice were imaged using the RFLSI‐Acquisition system with the mice positioned 40 cm below the detector. The focal length was adjusted to obtain clear blood flow images, and all photographs were taken at the same zoom (40x), exposure time (5 ms), and false‐color threshold Settings. RFSI images were analyzed by SIM BFI software. Mouse kidney rois were selected for quantitative perfusion. During RFLSI image acquisition and analysis, all examiners were unaware of the grouping of image mice.

### 2.5 NIR‐II imaging and analysis

Experimental mice were depilated on the abdomen, anesthetized with isoflurane gas, and their kidneys were surgically exposed. Subsequently, 200 μg (0.1 mg/mL) of ICG was injected into the tail vein of the imaged mice. NIR‐II fluorescence imaging was performed using an InGaAs SWIR camera (Xenics Cheetah‐640CL TE3) with a NIR‐II transmission lens (Spacecom VF50M SWIR). To optimally excite the ICG, an 808 nm laser was utilized as the excitation source with a power of 10,000 mW. To selectively capture the NIR‐II emission tail of the ICG dye,^35^, ^36^ the fluorescence signal was collected by a fixed long‐pass filter wheel (1100 nm, USA) at the front of the lens. The exposure time ranged from 0.5 to 2 s. After image acquisition, the contrast within the region of interest containing the kidney in NIR‐II imaging was quantified by calculating the cross‐sectional fluorescence intensity.

### 2.6 DUS imaging and analysis

Two‐dimensional ultrasound imaging, microcirculation perfusion imaging, and strain elastography (STE) were performed using a V700VE ultrasound system and an SL 3116 (12–25 MHz) probe (Esaote Medical Co., Ltd., China). Abdominal hair of mice was removed with depilation cream before ultrasound scanning, and then warm ultrasound gel was applied to the abdominal kidney region. For ultrasound imaging, mice were anesthetized with isoflurane and placed on the scanning platform, and physiological conditions such as heart and respiratory cycle were monitored, and B‐ultrasound, C‐ultrasound, and STE images were obtained.

### 2.7 CEUS imaging and analysis

Two‐dimensional ultrasound imaging and ultrasound microbubble imaging were performed with the ULTIMUS 9 LAB ultrasound system and a 10 to 23 MHz probe (VINNO, Suzhou, China). Abdominal hair of mice was removed with depilation cream before ultrasound scanning, and then warm ultrasound gel was applied to the abdominal kidney region. For ultrasound imaging, mice were anesthetized with isoflurane and placed supine on the scanning platform to monitor physiological conditions such as cardiac and respiratory cycles. Subsequently, 200 μg (0.1 mg/mL) of SF_6_ was injected into the tail vein of the imaged mice, and CEUS renal microvascular maps and renal microvascular perfusion maps were obtained by plane wave technique.

Following image acquisition, quantitative analysis of the microvascular networks was directly performed using the proprietary analytical tools integrated within the ultrasound imaging system. For each standardized region of interest (ROI), the software automatically extracts specific microvascular parameters based on the system's built‐in algorithms. (1) Vessel Ratio is defined as the spatial fraction occupied by the microvasculature, calculated using the formula: (Area of the vascular region within the ROI / Total Area of the ROI) × 100%, and is expressed as a percentage (%). (2) Mean Vascular Density reflects the average richness of localized blood perfusion. It is calculated by dividing the sum of vascular density values within the vascular region by the total number of pixels in that vascular region, expressed in arbitrary units (a.u.). (3) Vascular Complexity Level is an algorithm‐derived index evaluating the morphological, structural, and geometric disorder of the vascular image within the ROI. A higher complexity value indicates more irregular morphology, more complex structural filling in three‐dimensional space, and consequently, a more chaotic localized blood supply. The unit for the complexity level is expressed in arbitrary units (a.u.). (4) Perfusion Index serves as a comprehensive indicator of overall blood perfusion efficiency, calculated by multiplying the mean vascular velocity within the ROI by the vessel ratio, expressed in arbitrary units (a.u.).

### 2.8 Histopathological analysis

After anesthetizing the mice, the kidneys were immediately removed and cut in half. Each kidney was fixed in 4% formaldehyde overnight, then embedded in paraffin and sectioned into 5 μm thick slices. For morphological damage analysis, hematoxylin–eosin (HE), Masson staining, and periodic acid‐Schiff (PAS) staining were used. Pathological images were obtained using an optical microscope (Pannoramic MIDI, 3DHISTECH Ltd., Hungary). For quantitative morphometric analysis of the microvasculature, the absolute glomerular capillary luminal area was measured on PAS‐stained sections using ImageJ software (Version 1.53, NIH, USA). Briefly, 15–20 transverse glomerular profiles were randomly selected per animal. After calibrating the physical scale, images were converted to 8‐bit grayscale, and a consistent thresholding algorithm was applied within each glomerular profile to isolate and quantitatively calculate the absolute physical area (μm^2^) of the translucent capillary lumens. General histological changes in the mouse kidneys were determined, with 5 mice in each group and 5 slices examined for each animal. The accumulation and sclerosis of the renal glomerular lobule matrix (glomerular sclerosis index) was determined on PAS‐stained sections according to the following scoring system: 0: normal glomeruli; 1: matrix expansion or sclerosis involving up to 25% of the glomerular lobule; 2: sclerosis ranging from 25% to 50%; 3: sclerosis ranging from 50% to 75% and/or segmental glomerular extracellular fibrosis or proliferation; 4: global sclerosis >75%, global glomerular extracellular fibrosis or complete collapse of the glomerular lobule. The renal tubular injury markers are scored according to the following criteria: based on the percentage of cortical‐medullary junction showing renal tubular dilation, renal tubular atrophy, formation of renal tubular casts, vacuolation, degeneration, shedding of renal tubular epithelial cells or loss of brush border, and thickening of the renal tubular basement membrane: 0: none; 1: ≤10%; 2: 11–25%; 3: 26–45%; 4: 46–75%; 5: >76%.^37^ The kidney biopsy samples were evaluated by two independent nephrologists in a blinded manner.

### 2.9 Statistical analysis

Experimental data were expressed as mean ± SD or as individual values with medians based on the variable type. Statistical analysis was performed using GraphPad Prism 9 software. For comparisons between two independent groups, an unpaired Student *t*‐test was utilized for normally distributed data, whereas a Mann–Whitney – test was employed for data lacking normal distribution. For comparisons involving three or more groups, a one‐way analysis of variance (ANOVA) followed by a Tukey multiple comparisons test was utilized for normally distributed continuous variables. Crucially, a Kruskal–Wallis test followed by a Dunn multiple comparisons test was applied for ordinal histological grading data. A *p* value <0.05 was considered statistically significant.

It is important to note that the multimodal imaging parameters extracted in this study, including geometric area fractions (Vessel Ratio, Stiffness Area), dimensionless hemodynamic metrics (Doppler RI, branch‐to‐main Velocity Ratios), and intensive perfusion or signal properties (Mean Vascular Density, Perfusion Index, Mean Fluorescence/Speckle Intensity), are algorithmically normalized by spatial area or intrinsic temporal kinetics. Furthermore, a longitudinal tracking design was employed for the diseased cohorts to minimize inter‐individual baseline variability. Therefore, to prevent statistical confounding and artificial bias, post‐hoc mathematical normalization of these specific metrics to total body weight was neither required nor performed.

## 3 RESULTS

### 3.1 RFLSI imaging was used to evaluate the overall perfusion of the kidneys

The CKD model was constructed by 5/6 nephrectomy, while the DKD model was spontaneously established in db/db mice. Both models were continuously monitored using RFLSI. During the imaging process, the area around the kidneys was fully covered with sterile cotton balls to reduce the influence of blood flow from perirenal organs on renal imaging (Figure S1a). Macroscopic renal perfusion was quantified by extracting the relative ROI mean values (Figure S1b). In the CKD model, as the disease progressed, the macroscopic renal perfusion of the experimental mice gradually and continuously decreased (Figures 2a and S1c), showing a statistically significant difference compared with the CON group starting at 8 weeks (Figure 2c). Similarly, in the DKD model, the macroscopic renal perfusion also exhibited a progressive decline, decreasing significantly at 12 weeks compared with the db/m group, and deteriorating severely by 16 weeks (Figures 2b,d and S1c). This highly consistent decline across both models provides direct in vivo evidence of progressive macroscopic perfusion deficits driven by renal fibrosis. Furthermore, it is worth noting that after 8 weeks, the body weight of the CKD model mice decreased significantly, accompanied by increased irritability (Figure S3e).

**FIGURE 2:**
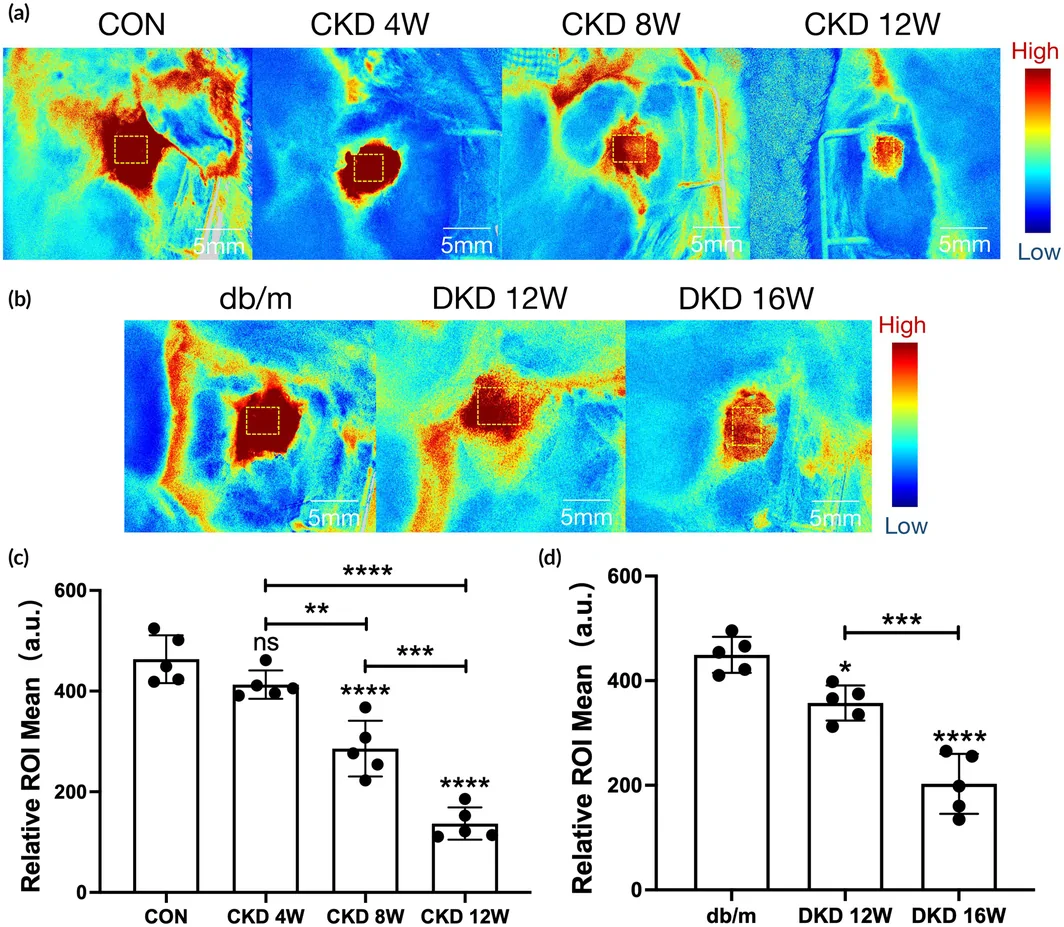
RFLSI imaging for monitoring renal perfusion of CKD and DKD models. (a, b) RFLSI imaging photos of the kidneys in the (a) CKD mice (CON, CKD 4W, CKD 8W, CKD 12W) and (b) DKD mice (db/m, DKD 12W, DKD 16W). The designated ROIs are outlined with dashed yellow lines, and the scale bar is 5 mm. (C, D) The relative ROI mean values (a.u.) of the (c) CKD and (d) DKD groups. There were 5 mice in each group (*n* = 5). *p < 0.05, **p < 0.01, ***p < 0.001, ****p < 0.0001; ns, not significant.

### 3.2 NIR‐II imaging shows the entire process of renal blood perfusion

To evaluate the dynamic changes in renal blood perfusion during the progression of RF, NIR‐II fluorescence imaging was performed immediately following the intravenous injection of ICG into the tail vein. Continuous monitoring successfully captured the entire perfusion process, which was recorded as dynamic time‐fluorescence intensity curves for the DKD model (Figures 3a and S2a, b) and the CKD model (Figures 3b and S2c, d). During the imaging process, the area around the kidneys was fully covered with sterile cotton balls to reduce the influence of blood flow from perirenal organs on renal imaging (Figure S1a). These kinetic curves clearly delineated three distinct physiological phases: the wash‐in phase, the peak phase, and the wash‐out phase. The minor physiological jaggedness observed in the raw curves was caused by the respiratory motion of the mice. Subsequently, we performed a comprehensive quantitative analysis of the fluorescence signals. In the DKD model, the mean fluorescence intensity, which represents the overall perfusion capacity, was significantly lower in the DKD mice compared to the db/m (Figure 3c). To further characterize the hemodynamic impairments, critical kinetic parameters were extracted. As DKD progressed, the time to peak was significantly prolonged (Figure 3d). Concurrently, both the wash‐in rate (Figure 3e) and the absolute wash‐out rate (Figure 3f) were significantly reduced in the DKD mice. Similar and profound hemodynamic alterations were also observed in the CKD model. Compared to the CON group, the mean fluorescence intensity showed a progressive decline as CKD advanced (Figure 3g). Similarly, the CKD mice exhibited a progressively delayed time to peak (Figure 3h), along with sharp, stage‐dependent decreases in both the wash‐in rate (Figure 3i) and the absolute wash‐out rate (Figure 3j). Interestingly, at 4 weeks in the CKD model, the time to peak and the wash‐in rate exhibited a slight reverse trend (although without statistical significance), which may be attributed to early compensatory mechanisms following nephrectomy. Collectively, these quantitative kinetic data robustly demonstrate that renal fibrosis leads to increased renal vascular resistance, sluggish inflow, and impaired clearance of the blood‐pool tracer. As renal fibrosis worsens, this significant decrease in perfusion could lead to further aggravation of hypoxia‐induced renal injury, thereby resulting in a vicious cycle.

**FIGURE 3:**
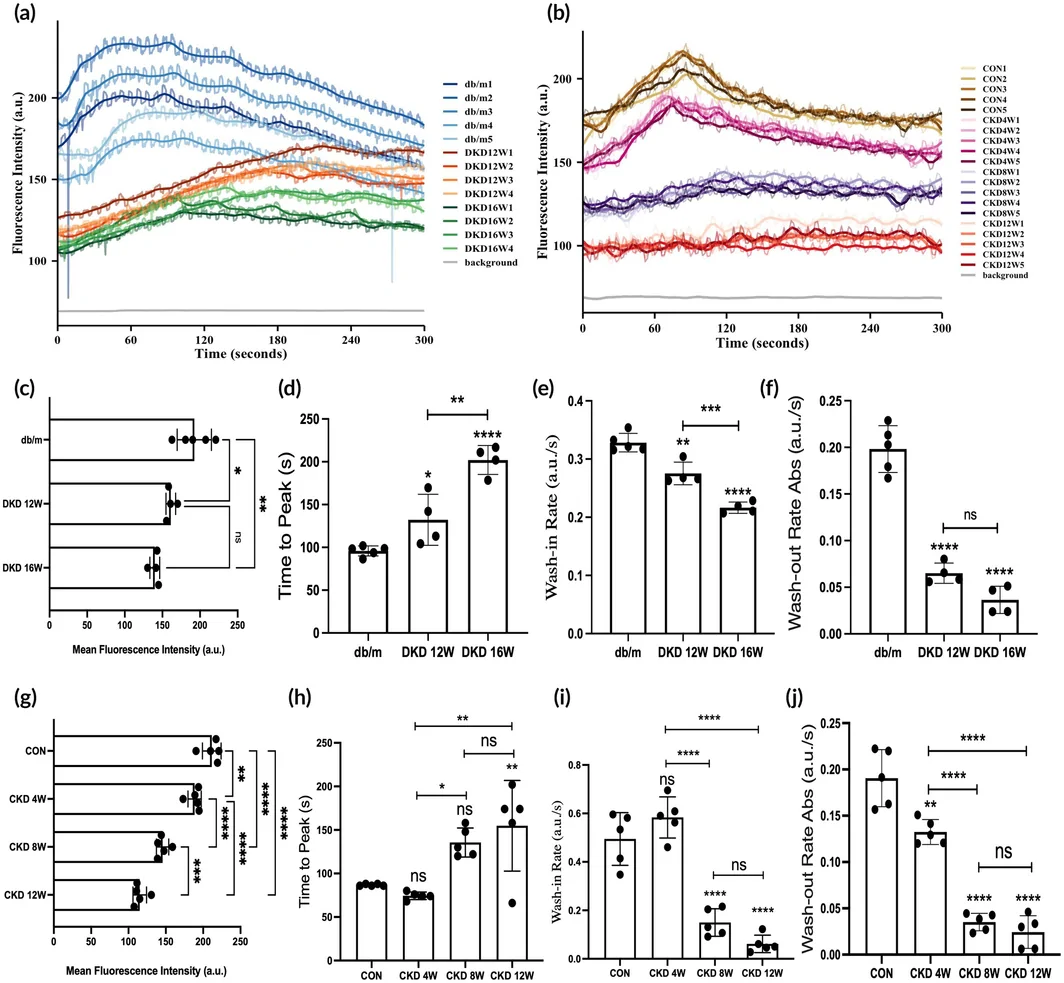
Quantitative NIR‐II fluorescence imaging for monitoring dynamic renal perfusion in DKD and CKD models. (a, b) Dynamic time‐fluorescence intensity curves tracking the entire renal perfusion process, monitored continuously for 300 s after ICG injection in (a) the DKD model (db/m, DKD 12W, DKD 16W) and (b) the CKD model (CON, CKD 4W, CKD 8W, CKD 12W). (C, G) Comparison of the mean fluorescence intensity (a.u.), representing overall renal perfusion capacity, in the (c) DKD and (G) CKD groups. (D, H) Statistical analysis of the time to peak (seconds) in the (d) DKD and (h) CKD models. (e, i) Quantification of the wash‐in rate (a.u./s) derived from the ascending phase of the perfusion curves for the (E) DKD and (i) CKD models. (f, j) The absolute wash‐out rate (a.u./s), calculated from the slope of the descending phase, evaluating the tracer clearance capability in the (f) DKD and (j) CKD models. db/m represents the non‐diabetic heterozygous littermate control group. Data are presented as mean ± SD (*n* = 4–5 per group). *p < 0.05, **p < 0.01, ***p < 0.001, ****p < 0.0001; ns, not significant.

### 3.3 DUS imaging shows the size, RI, microcirculation perfusion and hardness of the kidneys

Due to the much smaller diameter of blood vessels in mice compared to humans, customized ultrasound probes and microvascular imaging modes were utilized. First, we evaluated the general morphometrics of the mice. The kidney size was assessed using the longest diameter of the maximum transverse section. In the CKD model, the kidney size gradually decreased as the disease progressed (Figure S3a, c). Conversely, in the DKD model, the kidney size was significantly larger than that of normal mice at 12 weeks but significantly decreased by 16 weeks (Figure S3b, d). Consistent with these morphological changes, the body weight of DKD mice increased from 8 to 12 weeks and then showed a significant decline thereafter (Figure S3f). To further validate functional impairments and establish a robust link between structural remodeling and hemodynamic deficits, we evaluated the macroscopic vascular and physical properties of the kidneys. Recognizing that renal fibrosis is often accompanied by systemic vascular complications, we first measured the resistive index (RI) using pulsed‐wave Doppler ultrasound. In both the CKD and DKD models, the RIs of the juxtarenal abdominal aorta (reflecting systemic macrovascular stiffening) and main renal artery (reflecting downstream microvascular resistance) increased progressively as the disease advanced (Figure 4a,b). Finally, the structural microvasculature and tissue stiffness were assessed. High‐resolution color Doppler flow imaging revealed a significant decrease in renal microcirculation with disease progression (Figure 4c,d, top rows), which was quantitatively confirmed by a marked reduction in the Microvascular Perfusion Area (Figure 4e,f). Concurrently, ultrasound elastography demonstrated a progressive increase in kidney hardness, which serves as a biomechanical surrogate for fibrotic extracellular matrix deposition (Figure 4c,d, bottom rows; Figure 4g,h).

**FIGURE 4:**
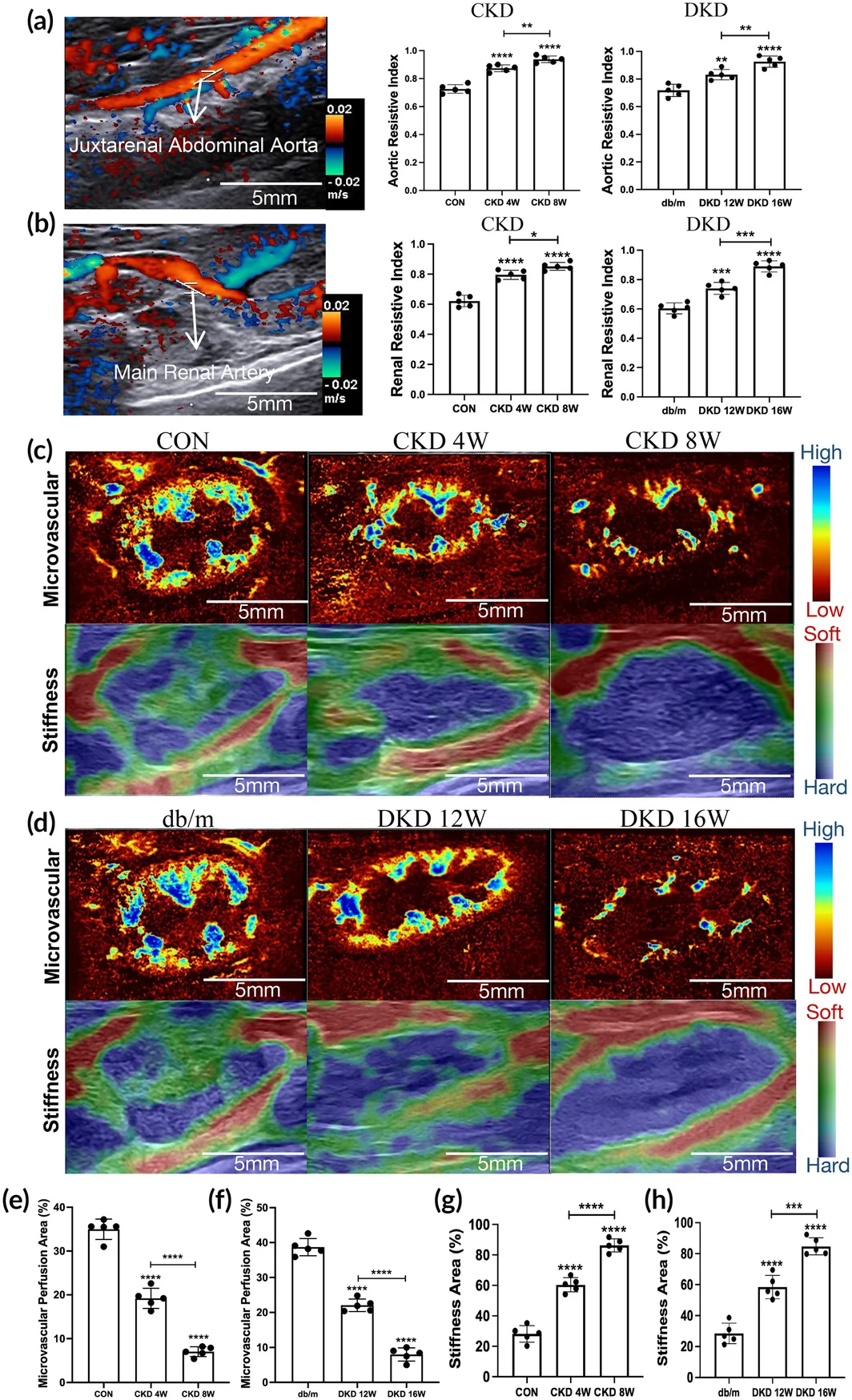
Ultrasound evaluation of renal hemodynamics, microvascular perfusion, and tissue stiffness in CKD and DKD mouse models. (a, b) Quantitative analysis of the abdominal aortic resistive index (RI) (a) and the main renal artery RI (b) across the CKD and DKD models. Scale bars = 5 mm. (c, d) Representative microvascular perfusion maps (top rows; color scale: Blue to red indicates high to low perfusion) and strain elastography (SE) images (bottom rows; color scale: Blue to red indicates hard to soft tissue) of the mouse kidneys in the CKD (c) and DKD (d) models. Scale bars = 5 mm. (e, f) Quantitative analysis of the microvascular perfusion area in the CKD (e) and DKD (f) models. (g, h) Quantitative analysis of the stiffness area in the CKD (g) and DKD (h) models. Data are presented as mean ± SD (*n* = 5 per group). *p <0.05, **p <0.01, ***p <0.001, ****p <0.0001, ns, not significant.

### 3.4 CEUS imaging angiography is used to show the density and complexity of renal microvessels

Sulfur hexafluoride (SF6) microbubbles were injected into CKD and DKD model mice via the tail vein for CEUS imaging (Figure 5a,b). In the CKD model, the complexity level, mean vascular density and vessel ratio of the renal microvasculature decreased significantly with the progression of renal fibrosis (Figure 5c,e). Strikingly, in the DKD model, the microvascular alterations exhibited parameter‐specific divergent trends at the early stage. At 12 weeks, the vascular complexity and vessel ratio of the experimental mice were significantly higher than those of normal mice, indicating early‐stage vascular bed expansion and pathological structural tortuosity. However, the mean vascular density at 12 weeks had already decreased significantly, suggesting an early decline in effective capillary perfusion quality despite the morphological expansion. As the disease progressed to 16 weeks, the renal vascular complexity, density, and vessel ratio all became significantly lower than those of the normal mice (Figure 5d,f), reflecting severe terminal capillary rarefaction. Microscopic quantification of the absolute glomerular capillary luminal area corroborated these CEUS findings (Figure S4). The CKD model showed a progressive collapse of the capillary lumens (Figure S4a). Conversely, the DKD model exhibited a compensatory vascular expansion at 12 weeks, structurally validating the elevated CEUS vessel ratio, followed by a severe microvascular collapse at 16 weeks (Figure S4b). Therefore, CEUS angiography can accurately and reliably monitor the dynamic progression of mouse kidney fibrosis by comprehensively evaluating these microvascular alterations.

**FIGURE 5:**
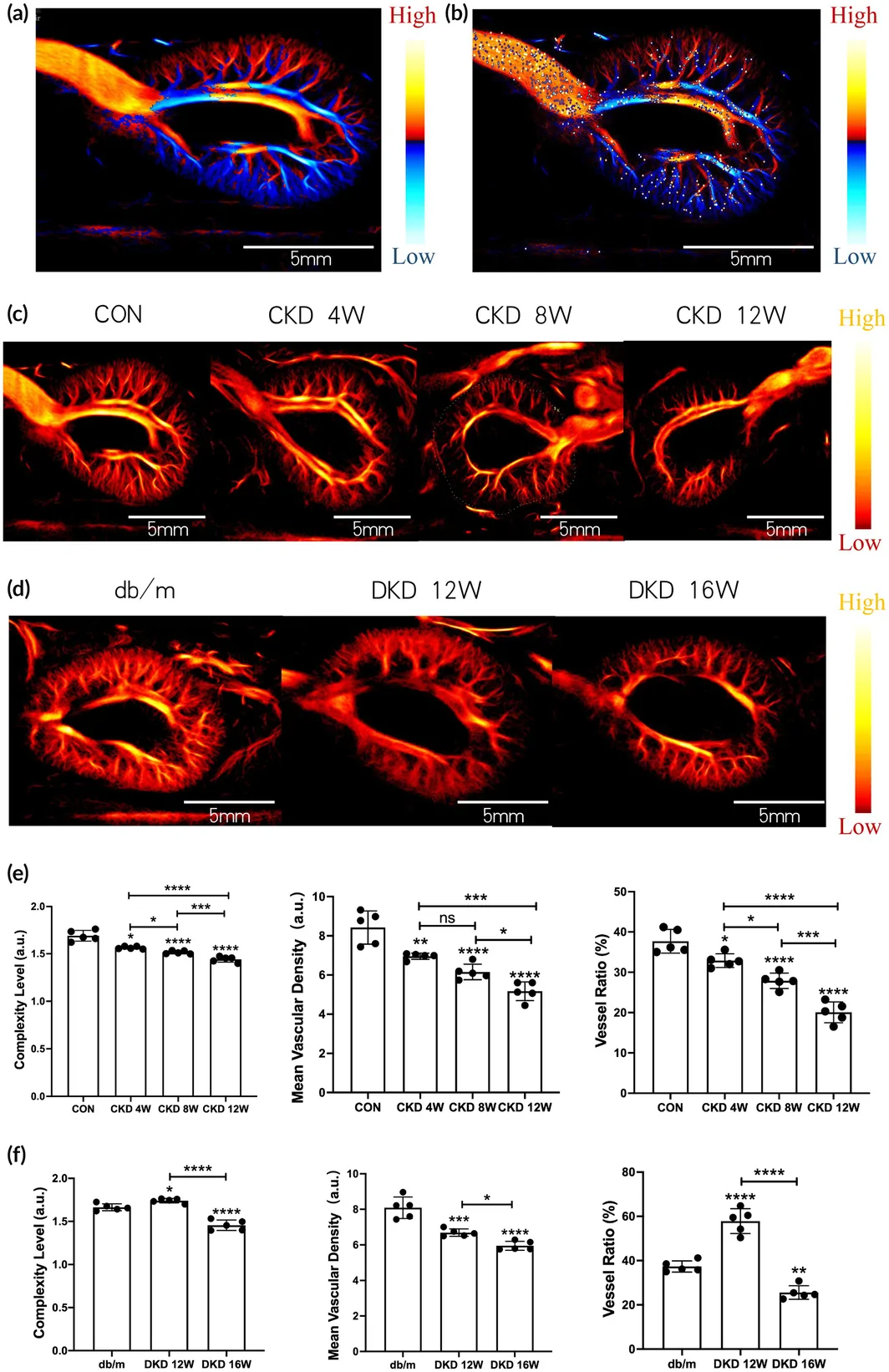
CEUS imaging for monitoring the density and complexity of the renal microcirculation of CKD and DKD mice. (a, b) Representative CEUS vascular image (The small dots in B represent microbubbles). (c, d) CEUS imaging was performed on the CKD (CON, 4W, 8W, 12W) and DKD (db/m, 12W, 16W) groups of mice. The detection indicators were: Vascular complexity (Complexity Level), vascular density (Mean Vascular Density), and vascular ratio (Vessel Ratio). There were 5 mice in each group. (e, f) Quantitative analysis of vascular complexity (Complexity Level), vascular density (Mean Vascular Density), and vascular ratio (Vessel Ratio) in CKD and DKD mice. *p <0.05, **p <0.01, ***p <0.001, ****p <0.0001, ns, not significant.

### 3.5 CEUS imaging perfusion maps are used to monitor renal perfusion and the blood flow velocity of vessels at all levels

According to the research of Katsuyuki Tanabe et al.,^38^ renal vessels are classified into five grades: Renal Artery, Segmental Arteries, Interlobar Arteries, Arcuate Arteries, Interlobular Arteries. In both the CKD and DKD models, the renal perfusion index decreased significantly with the progression of fibrosis. To minimize interindividual baseline variability and more accurately evaluate hemodynamic changes across different hierarchical levels of the renal vasculature, the blood flow velocity of each intrarenal branch was normalized to that of the main renal artery (Figure 6a,b). In both the CKD and DKD models, the velocity ratio of the primary branch to the main renal artery exhibited a progressive increase alongside disease advancement. This phenomenon is likely attributable to vascular stenosis occurring in the main renal artery proximal to these primary branches. Conversely, within the subsequent downstream vasculature, the blood flow velocity ratios relative to the main renal artery demonstrated a continuous decline as the disease progressed (Figure 6c–f). This downstream hemodynamic decline is likely driven by structural lesions, such as basement membrane thickening and mesangial matrix expansion within the renal microvasculature, particularly the glomerular capillaries, resulting in progressive luminal narrowing and occlusion.^39^ Consequently, these findings highlight the immense clinical translational value of CEUS in accurately profiling vascular hemodynamics across the entire renal arterial hierarchy.

**FIGURE 6:**
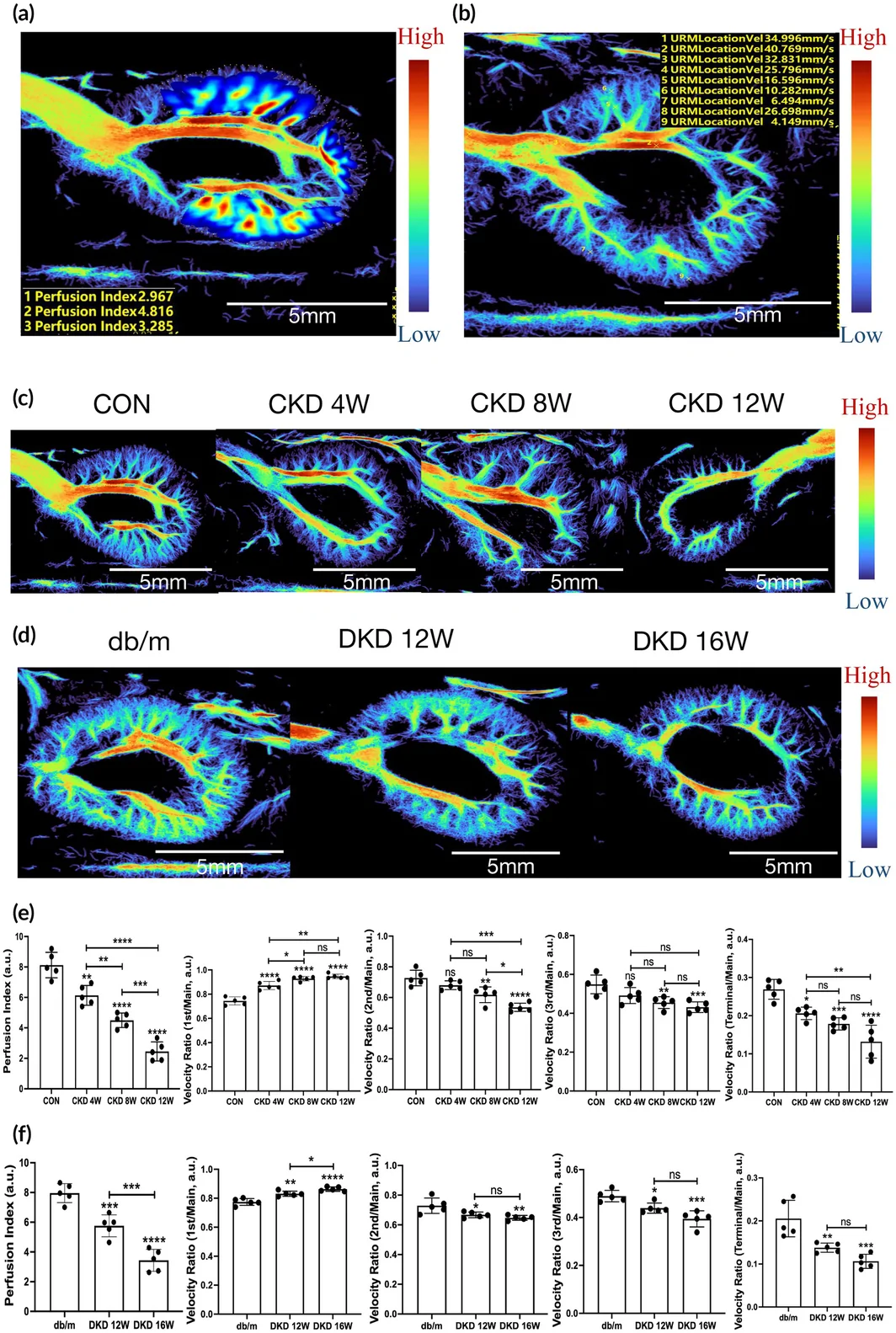
CEUS imaging for monitoring the perfusion and blood flow velocity of the renal microcirculation of CKD and DKD mice. (a, b) Representative CEUS perfusion image (a: Perfusion; b: Flow velocity). (c, d) CEUS imaging was performed on the CKD (CON, 4W, 8W, 12W) and DKD (db/m, 12W, 16W) groups of mice. The detection indicators were: Perfusion index (Perfusion Index), and Blood flow velocity (Renal main vessel, The first branch vessel, The second branch vessel, The third branch vessel, Terminal vessel). There were 5 mice in each group. (e, f) Quantitative analysis of perfusion index (Perfusion Index), The first branch vessel/Renal main vessel (1st/Main), The second branch vessel/Renal main vessel (2nd/Main), The third branch vessel/Renal main vessel (3rd/Main), and Terminal vessel/Renal main vessel (Terminal/Main) in CKD and DKD mice. *p <0.05, **p <0.01, ***p <0.001, ****p <0.0001, ns, not significant.

### 3.6 Comprehensive analysis of the degree of fibrosis in chronic kidney disease based on pathological features

Finally, we selected the kidneys of CKD model mice at 4 weeks, 8 weeks, 12 weeks as well as DKD model mice at 12 weeks, 16 weeks for pathological H&E, Masson and PAS staining. In CKD groups, as shown by Masson and PAS staining, the degree of renal fibrosis, glomerular sclerosis index and tubular injury gradually increase with the progression of CKD, and are negatively correlated with the density of renal microcirculation and the perfusion index of the kidney (Figure 7a,b). In the staining results, we also found that compared with the Sham group, the degree of renal fibrosis, glomerular sclerosis index, and renal tubular injury at CKD4w did not show significant changes, while the density of renal microcirculation and the perfusion index of the kidneys showed significant differences (Figures 5e and 6e). These results confirm that microvascular atrophy occurs earlier than tubular atrophy and interstitial collagen deposition during the progression of CKD. Similar findings were also observed in DKD. As DKD progressed, the degree of renal fibrosis, glomerular sclerosis index, and tubular damage gradually increased, and were negatively correlated with the density of renal microcirculation and the perfusion index of the kidneys (Figure 7c,d).

**FIGURE 7:**
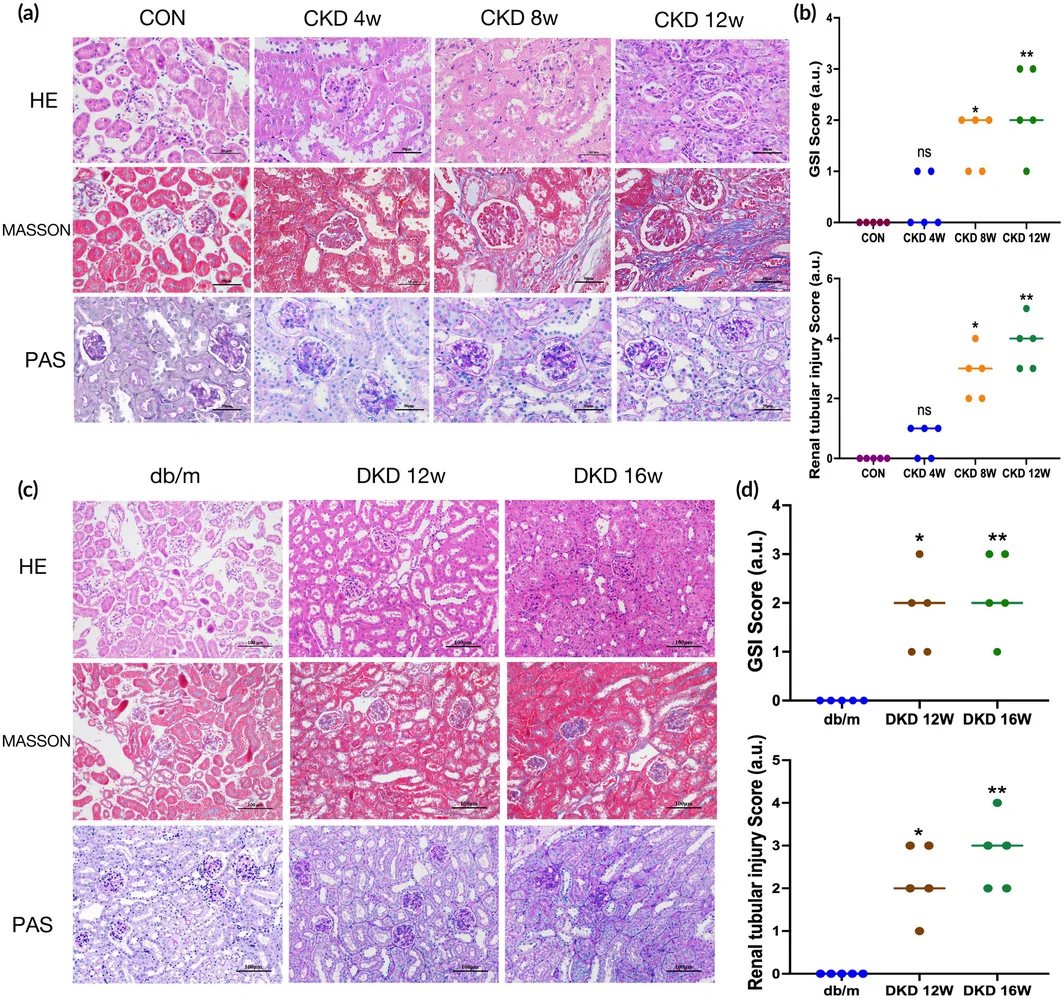
Histopathologic analysis of CKD and DKD models. (a, c) Representative micrographs of HE, Masson trichrome, and PAS staining of kidney sections from the CKD cohort (CON, CKD 4w, CKD 8w, CKD 12w) and the DKD cohort (db/m, DKD 12w, DKD 16w). (b, d) Quantitative statistical analysis of the histopathological grading, specifically GSI Score (a.u.) and Renal tubular injury Score (a.u.), corresponding to the CKD and DKD cohorts. Data are presented as individual values with the median indicated by a horizontal line. The sample size is *n* = 5 mice per group. *p <0.05, **p <0.01, ***p <0.001, ****p <0.0001, ns, not significant.

## 4 DISCUSSION

In this study, we established a stable and effective CKD and DKD model using five‐sixths nephrectomy and db/db mice. These two models represent the major causes of kidney fibrosis. For the first time, RFLSI, NIR‐II, US, and CEUS were used to comprehensively evaluate the entire process of renal blood perfusion, kidney size, hardness of the damaged kidney, hardness, microvascular density and complexity of the kidney, and blood flow velocity of all levels of blood vessels during the progression of renal fibrosis. The results were also verified by histopathology.

Renal fibrosis mainly refers to interstitial renal fibrosis, which is often accompanied by vascular occlusion, glomerulosclerosis, tubular atrophy, and arteriosclerosis.^40^ In the early stage of renal interstitial fibrosis, the permeability of interstitial capillaries increases, and the gradual reduction of interstitial capillary surface area leads to renal hypoxia and affects cell function.^1^ While renal biopsy provides definitive histopathology, its associated severe risks, such as bleeding and even death in a small number of patients,^41^ preclude its routine use for the long‐term monitoring of disease progression.^42^ To overcome this clinical bottleneck, obtaining finer and more comprehensive perfusion and vascular data is essential to dynamically assess the severity of CKD and DKD and to predict treatment response.

Laser Speckle Imaging (LSI) visualizes the microvasculature based on the dynamic scattering of laser light by moving red blood cells. Because it enables real‐time, high‐resolution in vivo monitoring without the need for tissue contact or exogenous tracers,^22^, ^43^ LSI is highly suitable for both clinical and experimental research. For example, Andrew P Carlson et al. reported the relative feasibility of preoperative vascular flow assessment using LSI to plan complex incision closure.^44^ Wido Heeman et al. discussed the use of LSI in laparoscopic surgery to identify ischemic bowel with microcirculation injury to reduce the incidence of anastomotic leakage.^45^ In this study, a comprehensive perfusion map of the renal cortex was provided by LSI, and the average LSI perfusion within the region of interest was quantified to show the perfusion changes in different stages of renal fibrosis. After the formation of renal fibrosis, the fluorescence intensity of the damaged kidney gradually decreased with the progress of renal fibrosis.

In recent years, NIR‐II imaging has become an emerging technology in comprehensive diagnosis and image‐guided therapy. With its advantages of high imaging resolution, low background, low autofluorescence, and deep tissue imaging ability, a variety of probes have been developed to identify microscopic breast cancer,^46^ hepatocellular carcinoma,^47^, ^48^ lung cancer,^49^ and high grade glioma^50^ and other lesions. In this study, NIR‐II nephrography with ICG was able to monitor the whole process of renal perfusion. In the mouse model, we observed that the maximum fluorescence intensity of the DKD group was significantly lower than that of the normal group, and the absolute value of the slope of the ascending segment and descending segment was significantly lower than that of the normal group, indicating that during the process of renal fibrosis due to vascular calcification, the renal blood vessels were narrowed, resulting in blood stasis in the kidney. This phenomenon suggests that renal fibrosis reduces renal blood flow and causes blood stasis in the kidney. This state of hypoxia will further aggravate renal fibrosis; if not interfered, it will form a vicious circle.

It is important to note the strong complementary nature of RFLSI and NIR‐II imaging in this study. While both are optical modalities assessing perfusion, they capture distinct dimensions of hemodynamics. RFLSI relies on endogenous hemoglobin absorption, providing a label‐free, steady‐state mapping of the renal cortical microcirculation. Conversely, NIR‐II utilizes an exogenous tracer (ICG) to trace the dynamic kinetics of the entire perfusion process, allowing us to mathematically calculate the slopes of wash‐in and wash‐out phases to identify deep‐tissue blood stasis. Therefore, RFLSI and NIR‐II cross‐validate each other by providing label‐free steady‐state mapping and tracer‐based dynamic kinetics, respectively.

Doppler ultrasound (DUS) remains a cornerstone imaging modality for evaluating CKD due to its non‐invasive nature, real‐time capability, cost‐effectiveness, and lack of ionizing radiation.^51^ However, conventional B‐mode ultrasound is largely restricted to gross morphological assessments and fails to capture subtle structural remodeling. To overcome this, strain elastography (SE) has emerged as a vital tool to evaluate tissue stiffness by quantifying tissue deformation under applied stress. As demonstrated by Gao et al., the elastic strain value of the renal cortex evaluated by SE is intimately correlated with the degree of renal interstitial fibrosis (IF).^28^ Furthermore, altered mechanical elasticity often heralds impaired regional perfusion.^52^ Building upon this, our study utilized a multiparametric ultrasound approach to evaluate not only the local renal microenvironment but also the associated systemic macrovascular hemodynamics. By simultaneously quantifying kidney stiffness and measuring the resistive indices (RIs) of both the juxtarenal abdominal aorta and main renal arteries, our study dynamically captured the concurrent progression of systemic macrovascular stiffening and renal hemodynamic deficits. Moreover, through microvascular imaging, we confirmed a stark negative correlation between renal microcirculation perfusion and fibrotic progression. Nonetheless, we acknowledge the inherent limitations of standard 2D Doppler ultrasound, which restricts the systematic, three‐dimensional, and deep‐tissue mapping of the complete microvascular hierarchical architecture.

There is a lack of diagnostic tools for evaluating renal perfusion in clinical work. It is difficult to achieve the expected evaluation standard by only relying on color Doppler flow imaging and pulse Doppler RI measurement. CEUS is a new imaging technology gradually developed in recent years and has gradually become the mainstream in the field of imaging. CEUS takes advantage of the strong reflective properties of microbubble contrast agents, which typically consist of a gas core and shell, under ultrasound to enhance the visualization of blood flow signals. These microbubbles can significantly improve the resolution and contrast of ultrasound images when flowing in blood, allowing clearer observation of the hemodynamic characteristics of organs and tissues.^29^, ^30^, ^31^ In our study, by comparing multi‐parametric microvascular metrics, we found that the density and complexity of blood vessels decreased significantly with the progression of renal fibrosis in the CKD model. Previous studies have shown that basement membrane thickening and extracellular matrix expansion compress and block the capillaries, directly leading to the decrease in vascular density and blood flow velocity.^39^, ^53^ Strikingly, early‐stage DKD (12 weeks) exhibited a unique apparent paradox in our ultrasonic metrics: while the morphometric vessel ratio and complexity significantly increased, the effective mean vascular density had already begun to decrease. These initially elevated metrics reflect compensatory vasodilation and abnormal neovascularization driven by hyperglycemia.^54^, ^55^, ^56^ However, the concurrent drop in vascular density (which evaluates the actual microbubble concentration) indicates that this morphological expansion does not equate to functional hyperperfusion. Instead, structural compression from early extracellular matrix expansion, combined with endothelial dysfunction, increases downstream microcirculatory resistance and causes sluggish blood flow or stagnation within the dilated vascular beds.^57^ Our multiparametric ultrasound approach thus successfully uncouples structural dilation from true functional perfusion deficits. Interestingly, we found that the flow velocity ratio of the first branch to the main artery was directly proportional to the progression of fibrosis, while the velocity ratio of the second branch was inversely proportional to the progression of fibrosis, suggesting that the vascular lesions were most severe before the first branch and in the terminal vessels. This finding is highly consistent with clinical observations in CKD patients, where vascular lesions primarily involve the large and terminal vessels, underscoring its great clinical significance.^58^, ^59^, ^60^ Meanwhile, through supplementary verification using histopathological HE, Masson and PAS staining, we found that when ultrasound showed a significant decrease in renal microcirculation density and renal perfusion index in the early stage of renal fibrosis, there were no significant changes in the degree of histopathological renal fibrosis, glomerular sclerosis index, and renal tubular damage. This reflects the significant importance of monitoring renal microcirculation in diagnosing early renal fibrosis.

In our previous work, we successfully utilized non‐invasive multimodal imaging platforms to evaluate superficial vascular alterations and regeneration in acute lower limb ischemia.^61^ Building upon this methodological foundation, the current study transitions to addressing the deep‐tissue, chronic microcirculatory challenges inherent to renal fibrosis. By employing a distinct combination of macroscopic optical tracking (RFLSI, NIR‐II) and advanced ultrasonic (DUS, CEUS) modalities, we uniquely captured the progressive functional perfusion deficits in CKD and DKD models, effectively overcoming the depth limitations of our previous superficial imaging approaches.

Given the critical diagnostic value of microcirculation highlighted above, it is essential to contextualize our multimodal framework against prevalent clinical tools. While advanced modalities such as DCE‐MRI and DWI can provide extensive kinetic information regarding vascular perfusion,^62^ our approach offers distinct advantages tailored for early‐stage CKD/DKD assessment (Table 1).

Firstly, our framework prioritizes safety in the context of compromised renal function. A critical bottleneck for dynamic contrast enhanced MRI and CTA in CKD or DKD patients is the risk of complications induced by contrast media. Agents utilizing gadolinium in MRI pose a fatal risk of nephrogenic systemic fibrosis (NSF),^63^ while iodinated CT contrast agents can trigger contrast induced nephropathy (CIN).^64^ Furthermore, these agents often extravasate into the fibrotic interstitium, confounding perfusion quantification.^62^ Our strategy avoids these risks by utilizing CEUS with inert gas microbubbles. These microbubbles function strictly as intravascular blood pool agents and are eliminated exclusively via pulmonary exhalation, imposing zero metabolic or filtration burden on the kidneys. Secondly, the integration of DUS and CEUS offers superior clinical feasibility and cost efficiency. Unlike the high operational costs and specialized infrastructure required for MRI or CT, ultrasonic techniques are compatible with point of care diagnostics and are completely free of radiation. This tiered diagnostic strategy, utilizing DUS for baseline macrohemodynamics and CEUS for functional microperfusion, provides an affordable pathway completely free of nephrotoxicity for early detection and longitudinal monitoring.

Despite the significant translational potential of this multimodal approach, several limitations in our study must be acknowledged. First, the current study is inherently limited by its relatively small sample size, which may constrain the statistical power and broad generalizability of our findings. Specifically, this sample size constraint limited the statistical stability of our linear mixed‐effects models (LMMs) when evaluating multiple predictive parameters simultaneously (Figure S5), particularly in the DKD cohort. Consequently, this initial investigation and our exploratory multi‐marker modeling should be interpreted as a proof‐of‐concept demonstrating the synergistic diagnostic potential of multimodal CEUS parameters. Second, while we objectively demonstrated the renal vascular changes during the progression of renal fibrosis in CKD and DKD mice, the specific molecular mechanisms underlying the crosstalk between vascular lesions and fibrogenesis remain unexplored.^65^ Third, the multimodal imaging devices and specific acquisition procedures require further standardization to yield reproducible and accurate perfusion parameters, which is a prerequisite for widespread clinical promotion.

In summary, while many of the imaging technologies described above are still in their early stages of clinical translation, their combination offers unprecedented opportunities. Macroscopic optical techniques provide comprehensive mapping of cortical hemodynamics, but their direct clinical application for internal organs is currently restricted by the need for surgical exposure. Conversely, noninvasive acoustic imaging, particularly CEUS, is highly sensitive to functional microperfusion and effectively overcomes the depth penetration limits of optical techniques. Their synergistic combination enables comprehensive visualization and quantification of renal hemodynamics. CEUS, in particular, not only delineates vessel location, size, and morphology but also dynamically assesses blood flow velocity, direction, and tissue perfusion. Moving forward, expanding the cohort size to validate these preliminary findings and ultimately incorporating nonlinear machine learning algorithms to construct a robust, independently validated multiparametric diagnostic model represent the primary objectives of our future research. Ultimately, CEUS could significantly enhance our ability to noninvasively detect the severity of renal fibrosis and continuously track disease progression, thereby greatly facilitating clinical decision making.

## 5 CONCLUSIONS

In conclusion, this study pioneers a multimodal imaging framework combining RFLSI, NIR‐II, DUS, and CEUS to comprehensively monitor renal perfusion and vascular hemodynamics during fibrotic progression. Among these modalities, CEUS demonstrated exceptional advantages in functional microvascular imaging, providing a noninvasive and quantitative strategy for monitoring renal perfusion, microcirculatory neovascularization, and blood flow velocities across the entire renal arterial hierarchy. Consequently, CEUS holds immense clinical promise for the dynamic diagnosis and longitudinal tracking of renal fibrosis, as well as for evaluating therapeutic efficacy and patient prognosis. Ultimately, this multiparametric ultrasonic approach is poised to become a pivotal clinical tool for the early screening and precise management of renal fibrosis.

**Abbreviations**
CEUS: contrast‐enhanced ultrasound
CKD: Chronic kidney disease
DKD: Diabetic kidney disease
DUS: duplex ultrasound
ESRD: end‐stage renal disease
FDA: Food and Drug Administration
GFR: glomerular filtration rate
HE: Hematoxylin and Eosin
ICG: Indocyanine green
NIR‐II: near‐infrared II imaging
PAS: periodic acid‐Schiff
RAAS: renin‐angiotensin‐aldosterone system
RF: Renal fibrosis
RFLSI: real‐time and wide‐field laser speckle imaging
SE: Strain Elastography
SF_6_
: Sulfur Hexafluoride
W: weeks

## AUTHOR CONTRIBUTIONS

**Yijie Ning:** Conceptualization; validation; methodology; formal analysis; visualization. **Yuhang Zhang:** Conceptualization; methodology; software; data curation; resources; formal analysis; validation; visualization; investigation; writing – original draft; writing – review and editing. **Chuanlong Lu:** Data curation; validation; investigation. **Qian Wang:** Conceptualization; methodology; validation; formal analysis; visualization. **Yaling Li:** Conceptualization; validation; software; visualization. **Honglin Dong:** Conceptualization; investigation; funding acquisition; writing – review and editing; visualization; validation; methodology; software; formal analysis; project administration; resources; supervision; data curation. **Zeyu Zhang:** Methodology; validation; visualization; formal analysis; data curation. **Ruijing Zhang:** Conceptualization; methodology; software; data curation; supervision; investigation; validation; visualization; formal analysis; project administration; resources; writing – review and editing; writing – original draft; funding acquisition. **Xiaohua Jia:** Data curation; formal analysis; visualization; validation; investigation. **Heng Wang:** Conceptualization; methodology; validation; data curation; visualization. **Keyi Fan:** Methodology; validation; visualization; formal analysis.

## FUNDING INFORMATION

This work received the support from the Regional Cooperation Program of Shanxi Province, China (Grant No. 202204041101038); The Regional Cooperation Program of Shanxi Province, China (Grant No. 202504041101034); The Leading Talent Team Building Program of Shanxi Province, China (Grant No. 202204051002010); Construction and demonstration of molecular diagnosis and treatment platform for vascular diseases in Shanxi Province, China (Grant No. SCP‐2023‐17); Translational Medicine Engineering Research Center for Vascular Diseases of Shanxi Province, China (Grant No. 2022017); The Shanxi Provincial Science and Technology Department Centralized Guided Local Projects (Grant No. YDZJSX2021C026); The National Natural Science Foundation of China (Grant No. 81770695, 81870354).

## CONFLICT OF INTEREST STATEMENT

The authors have no conflicts of interest to declare.

## Supporting information


**FIGURE S1.** RFLSI imaging of renal microcirculation in CKD and DKD models. (a) The physical diagram of the method for imaging the RFLSI of mouse kidneys. (b) The change in fluorescence intensity of the region of interest during RFLSI imaging. (c) RFLSI perfusion heatmaps of mouse kidneys in the CKD cohort across different stages, including CON, CKD 4W, CKD 8W, and CKD 12W. (d) RFLSI perfusion heatmaps of mouse kidneys in the DKD cohort, including db/m, DKD 12W, and DKD 16W. The color scale qualitatively represents the relative blood perfusion levels ranging from High to Low. Scale bars represent 5 mm. There were 5 mice in each group.
**FIGURE S2.** Continuous monitoring of renal perfusion in CKD and DKD models using NIR‐II imaging. (a, c) Representative NIR‐II fluorescence images of mouse kidneys from the CKD cohort (CON, CKD 4W, CKD 8W, CKD 12W) and the DKD cohort (db/m, DKD 12W, DKD 16W), captured at 30 s, 1 min, 3 min, and 5 min following intravenous ICG injection. (b, d) Quantitative analysis of ICG signal intensity, expressed as ROI mean fluorescence, corresponding to the CKD and DKD cohorts over the monitored time points. Data are presented as mean ± SD (*n* = 4–5 per group).
**FIGURE S3.** DUS imaging of macroscopic kidney dimensions and longitudinal body weight changes. (a, b) Representative B‐mode ultrasound images displaying the transverse diameter of kidneys in the (a) CKD group (CON, CKD 4W, CKD 8W) and (b) DKD group (db/m, DKD 12W, DKD 16W). The grayscale bar indicates ultrasound echogenicity (High/Low). (c, d) Quantitative analysis of the transverse diameter of the kidney (cm) in the (c) CKD and (d) DKD mice. (e, f) Quantitative analysis of systemic body weight (g) progression in the (e) CKD and (f) DKD mice. There were five mice in each group (*n* = 5). Statistical significance: ns, not significant; *p <0.05, **p <0.01, ***p <0.001, ****p <0.0001.
**FIGURE S4.** Quantitative analysis of the absolute glomerular capillary luminal area. (a, b) Quantitative analysis of the absolute capillary luminal area (μm^2^) based on PAS‐stained sections in the (a) CKD mice (CON, CKD 4W, CKD 8W, CKD 12W) and (b) DKD mice (db/m, DKD 12W, DKD 16W).
**FIGURE S5.** Exploratory analysis of Linear Mixed‐Effects Models (LMMs) integrating multimodal imaging parameters. (a) Standardized forest plot of the full multivariable LMM incorporating Doppler RI, Tissue Stiffness, CEUS‐DEN, and CEUS‐PI. (b) Variance Inflation Factor (VIF) analysis revealing severe multicollinearity (VIF >10 for PI, RI, and Stiffness), leading to inflated parameter uncertainty. (c) Spearman correlation matrix demonstrating profound inter‐parameter redundancy between macroscopic and microcirculatory indices. (d) Standardized forest plot of the optimized model utilizing only microvascular parameters (DEN and PI), showing independent predictive value. (e) VIF analysis confirming the resolution of collinearity (VIF <10). (f) Receiver operating characteristic (ROC) curves demonstrating that the combined multiparametric model (AUC = 0.96) significantly outperforms single‐parameter models (AUC = 0.92 for both DEN alone and PI alone). (g) Akaike Information Criterion (AIC) lollipop chart confirming superior model fit for the combined approach (lowest AIC = −3.06) via likelihood ratio testing (*p* <0.005). (h) Standardized forest plot and (i) VIF analysis of the four‐parameter model showing persistent, severe multicollinearity. (j) Spearman correlation matrix for the DKD cohort. (k) VIF analysis of the two‐parameter (DEN + PI) model in DKD, indicating that collinearity and parameter uncertainty remain unacceptably high even after feature reduction.

## Data Availability

The datasets generated and analyzed during the current study are available from the corresponding author upon reasonable request.
