# Supplementary material for: Monitoring vascular changes in renal fibrosis in mice using multimodal optical and ultrasonic imaging
Source: Bioeng Transl Med. 2026 Aug 9;11(5):e70169. doi: 10.1002/btm2.70169 (PMC13454415; doi:10.1002/btm2.70169)
Supplement: Supplementary file 1 [file BTM2-11-e70169-s001.docx]

**Supplementary Figure Legends**

**Figure S1: RFLSI imaging of renal microcirculation in CKD and DKD models.**

(A) The physical diagram of the method for imaging the RFLSI of mouse kidneys. (B) The change in fluorescence intensity of the region of interest during RFLSI imaging. (C) RFLSI perfusion heatmaps of mouse kidneys in the CKD cohort across different stages, including CON , CKD 4W , CKD 8W , and CKD 12W. (D) RFLSI perfusion heatmaps of mouse kidneys in the DKD cohort, including db/m , DKD 12W , and DKD 16W. The color scale qualitatively represents the relative blood perfusion levels ranging from High to Low. Scale bars represent 5mm. There were 5 mice in each group.

**Supplementary Figure 2. Continuous monitoring of renal perfusion in CKD and DKD models using NIR-II imaging.**

(A, C) Representative NIR-II fluorescence images of mouse kidneys from the CKD cohort (CON, CKD 4W, CKD 8W, CKD 12W) and the DKD cohort (db/m, DKD 12W, DKD 16W), captured at 30 seconds, 1 minute, 3 minutes, and 5 minutes following intravenous ICG injection. (B, D) Quantitative analysis of ICG signal intensity, expressed as ROI mean fluorescence, corresponding to the CKD and DKD cohorts over the monitored time points. Data are presented as mean ± SD (n = 4–5 per group).

**Figure S3: DUS imaging of macroscopic kidney dimensions and longitudinal body weight changes. (A, B)** Representative B-mode ultrasound images displaying the transverse diameter of kidneys in the **(A)** CKD group (CON, CKD 4W, CKD 8W) and **(B)** DKD group (db/m, DKD 12W, DKD 16W). The grayscale bar indicates ultrasound echogenicity (High/Low). **(C, D)** Quantitative analysis of the transverse diameter of the kidney (cm) in the **(C)** CKD and **(D)** DKD mice. **(E, F)** Quantitative analysis of systemic body weight (g) progression in the **(E)** CKD and **(F)** DKD mice. There were 5 mice in each group (n=5). Statistical significance: ns, not significant; *p < 0.05, **p < 0.01, ***p < 0.001, ****p < 0.0001.

**Figure S4: Quantitative analysis of the absolute glomerular capillary luminal area.**

**(A, B)** Quantitative analysis of the absolute capillary luminal area (μm²) based on PAS-stained sections in the **(A)** CKD mice (CON, CKD 4W, CKD 8W, CKD 12W) and **(B)** DKD mice (db/m, DKD 12W, DKD 16W).

**Figure S5: Exploratory analysis of Linear Mixed-Effects Models (LMMs) integrating multimodal imaging parameters.**

(A) Standardized forest plot of the full multivariable LMM incorporating Doppler RI, Tissue Stiffness, CEUS-DEN, and CEUS-PI. **(B)** Variance Inflation Factor (VIF) analysis revealing severe multicollinearity (VIF > 10 for PI, RI, and Stiffness), leading to inflated parameter uncertainty. **(C)** Spearman correlation matrix demonstrating profound inter-parameter redundancy between macroscopic and microcirculatory indices. **(D)** Standardized forest plot of the optimized model utilizing only microvascular parameters (DEN and PI), showing independent predictive value. **(E)** VIF analysis confirming the resolution of collinearity (VIF < 10). **(F)** Receiver operating characteristic (ROC) curves demonstrating that the combined multiparametric model (AUC = 0.96) significantly outperforms single-parameter models (AUC = 0.92 for both DEN alone and PI alone). **(G)** Akaike Information Criterion (AIC) lollipop chart confirming superior model fit for the combined approach (lowest AIC = -3.06) via likelihood ratio testing (P < 0.005). **(H)** Standardized forest plot and **(I)** VIF analysis of the four-parameter model showing persistent, severe multicollinearity. **(J)** Spearman correlation matrix for the DKD cohort. **(K)** VIF analysis of the two-parameter (DEN + PI) model in DKD, indicating that collinearity and parameter uncertainty remain unacceptably high even after feature reduction.
